# Supplementary material for: Exact Discrete Stochastic Simulation With Deep‐Learning‐Scale Gradient Optimization
Source: Adv Sci (Weinh). 2026 Jul 10:e76297. Online ahead of print. doi: 10.1002/advs.76297 (PMC13353176; doi:10.1002/advs.76297)
Supplement: Supplementary file 1 — Supporting File: advs76297‐sup‐0001‐SuppMat.pdf. [file ADVS-9999-e76297-s001.pdf]

Exact Discrete Stochastic Simulation with Deep-Learning-Scale Gradient Optimization

(Supporting Information)

Jose M. G. Vilar[1,2,*] and Leonor Saiz[3,*]

[1] Biofisika Institute (CSIC, UPV/EHU), University of the Basque Country (UPV/EHU), P.O. Box 644, 48080 Bilbao, Spain

[2] IKERBASQUE, Basque Foundation for Science, 48011 Bilbao, Spain

[3] Department of Biomedical Engineering, University of California, 451 E. Health Sciences Drive, Davis, CA 95616, USA

[*]Correspondence to: j.vilar@ikerbasque.org or lsaiz@ucdavis.edu

Note S1: Score-function estimators and alternative gradient-estimation strategies

Two alternative gradient-estimation strategies are theoretically applicable to the CTMC parameter-inference problem: score-function (REINFORCE-type) estimators [1] and parameter-by-parameter sensitivity methods [2, 3]. Both are unbiased or near-unbiased in principle. Neither is practical at deep-learning scales for the reasons developed below.

S1.1 The score-function estimator for CTMCs

A full CTMC sample path is a sequence of states, reaction indices, and waiting times,

$$\omega_{0:n} = (\boldsymbol{X}_0, J_0, \tau_0, \dots, J_{n-1}, \tau_{n-1}),$$

with deterministic updates $\boldsymbol{X}_{k+1} = \boldsymbol{X}_k + \boldsymbol{\nu}_{J_k}$. The Gillespie law gives the path measure

$$p_{\boldsymbol{\theta}}(\omega_{0:n}) = p(\boldsymbol{X}_0) \prod_{k=0}^{n-1} a_{J_k}(\boldsymbol{X}_k; \boldsymbol{\theta}) \exp[-a_0(\boldsymbol{X}_k; \boldsymbol{\theta})\, \tau_k],$$

since at step k the next reaction has probability density $a_{J_k} \exp(-a_0 \tau_k)$ for the joint event (firing reaction J_k at waiting time τ_k). The score-function gradient identity [1] then gives

$$\nabla \mathcal{L}(\boldsymbol{\theta}) = \mathbb{E}_{\boldsymbol{\theta}}\left[\ell(\omega_{0:n}) \sum_{k=0}^{n-1} \left(\nabla_{\boldsymbol{\theta}} \log a_{J_k}(\boldsymbol{X}_k; \boldsymbol{\theta}) - \tau_k\, \nabla_{\boldsymbol{\theta}} a_0(\boldsymbol{X}_k; \boldsymbol{\theta}) \right) \right].$$

This is unbiased: a single trajectory yields an unbiased estimate. The two contributions per step — the score of the chosen reaction and a waiting-time correction proportional to the gradient of the total propensity — are both analytically differentiable in $\boldsymbol{\theta}$. The score is therefore well-defined; variance is the binding constraint.

S1.2 Variance scaling with trajectory length

Define the per-step score $\boldsymbol{s}_k(\boldsymbol{\theta}) = \nabla_{\boldsymbol{\theta}} \log a_{J_k}(\boldsymbol{X}_k; \boldsymbol{\theta}) - \tau_k \nabla_{\boldsymbol{\theta}} a_0(\boldsymbol{X}_k; \boldsymbol{\theta})$ and the cumulative score $\boldsymbol{S}_n = \sum_{k=0}^{n-1} \boldsymbol{s}_k$, so the REINFORCE estimator is $\boldsymbol{g}_{\text{RF}} = \ell(\omega_{0:n}) \boldsymbol{S}_n$.

Proposition S1.1 (covariance accumulation for the trajectory score). *Under the trajectory measure, the per-step scores satisfy* $\mathbb{E}[\boldsymbol{s}_k \mid \boldsymbol{X}_k] = \mathbf{0}$ *for all* k. *The per-step contributions are therefore uncorrelated, and the trajectory-score covariance accumulates additively:*

$$\text{Cov}(\boldsymbol{S}_n) = \sum_{k=0}^{n-1} \text{Cov}(\boldsymbol{s}_k).$$

Proof. The conditional mean-zero property is the standard score identity: $\mathbb{E}[\nabla_{\boldsymbol{\theta}} \log p_{\boldsymbol{\theta}}(\omega_{k+1} \mid \boldsymbol{X}_k) \mid \boldsymbol{X}_k] = \nabla_{\boldsymbol{\theta}} \mathbb{E}[1 \mid \boldsymbol{X}_k] = \mathbf{0}$, where $\omega_{k+1} = (J_k, \tau_k)$ is the next reaction-index/waiting-time pair. The cross-term identity $\mathbb{E}[\boldsymbol{s}_k^\top \boldsymbol{s}_{k'}] = 0$ for $k \neq k'$ follows by tower-property conditioning on $\boldsymbol{X}_{\max(k,k')}$. Summing then yields the additive covariance decomposition. ■

The cumulative trajectory score therefore has covariance that grows additively with the number of events: whenever the per-step score covariances remain bounded below in some appropriate sense (e.g., bounded below in operator norm across non-absorbing states), the trace of $\text{Cov}(\boldsymbol{S}_n)$ grows linearly in n. The REINFORCE estimator $\boldsymbol{g}_{\text{RF}} = \ell\, \boldsymbol{S}_n$ multiplies this cumulative score by the trajectory loss, and its variance commonly inherits the same unfavorable trajectory-length scaling, unless strong variance reduction or special loss structure (e.g., losses that decorrelate from $\boldsymbol{S}_n$) is present. This phenomenon is documented across decades of work on discrete latent-variable models (variational autoencoders with categorical latents, structured prediction, and reinforcement learning [4-6]) and is the binding constraint for the deep regime considered here. For the MNIST classification network in particular ($n = 2{,}880$ steps, $p \approx 2 \times 10^5$ parameters), the cumulative-score covariance places standard REINFORCE well outside any practical compute budget by simple extrapolation from the established literature.

S1.3 Comparison with alternative estimators

Parameter-by-parameter sensitivity methods such as the Poisson Path Algorithm [2] and common-random-number finite differences [3] do not have the variance problem but introduce a per-parameter cost. The trade-offs are summarized below.

Method	Bias	Variance	Extra simulations per gradient estimate
REINFORCE (score function)	Unbiased	Additive accumulation (Prop. S1.1); linear in n under mild conditions	$\Theta(1)$
CRN finite differences	$O(h^2)$	$O(1/h^2)$	$\Theta(p)$
Poisson Path Algorithm	Unbiased	Often substantially reduced relative to standard REINFORCE; problem-dependent	$\Theta(p)$ for full gradient

Soft-forward Gillespie	Forward bias	Bounded	$\Theta(1)$
**Straight-through (this work)**	Surrogate bias (Note S2.3)	Per-step Jacobian bounded (spectral norm $\leq 1/(2T)$, Note S2.3); no trajectory-score product	$\Theta(1)$ (reverse-mode, not parameter-by-parameter)

For the dimerization ($p = 2$), oscillator ($p = 5$), and ion channel ($p = 3$) systems, the parameter-by-parameter methods remain practical and would yield comparable parameter recovery. For the MNIST classification network ($p \approx 2 \times 10^5$), the linear-in-p simulation cost of PPA and CRN finite differences increases the compute burden per gradient estimate by orders of magnitude relative to the reverse-mode ST framework, making a full MNIST-scale training run with these methods infeasible under the compute budget used here.

The straight-through estimator avoids the trajectory-score product of REINFORCE while retaining parameter-independent reverse-mode scaling. It pays for this with a surrogate bias whose variance is controlled empirically by ensemble averaging and temperature annealing, as the experiments in the main text and the temperature ablation in Figure S1 demonstrate. The soft-forward Gillespie [7] also achieves $\Theta(1)$ scaling but introduces a *forward* bias by relaxing the dynamics themselves, which our framework avoids by the forward-exactness result of Section 2.3 of the main text.

Note S2: Mathematical Appendix

At each CTMC step, exact Gillespie simulation requires a categorical draw over reaction channels. The Gumbel-Max construction represents this draw exactly, while the Gumbel-Softmax relaxation supplies a differentiable surrogate for the backward pass. The following results formalize this forward/backward decoupling and distinguish the exactness of the simulated trajectory from the bias of the surrogate gradient.

Throughout, M denotes the number of competing reaction channels, $\boldsymbol{X} \in \mathbb{Z}^N$ the state of the chemical system, $\boldsymbol{\theta} \in \mathbb{R}^p$ the parameter vector, and $a_j(\boldsymbol{X}; \boldsymbol{\theta}) \geq 0$ the propensity of reaction j, with $a_0 = \sum_k a_k > 0$ on every non-absorbing state. Channels with $a_j = 0$ are assigned $\log a_j = -\infty$ and therefore cannot be selected by the Gumbel-Max rule. The derivations below are written for the strictly positive case for notational simplicity; the nonnegative case follows by restricting the argmax and the softmax to the active set $\{j: a_j > 0\}$. We write $\pi_j = a_j/a_0$ for the normalized categorical probabilities, and $G_j \overset{\text{i.i.d.}}{\sim} \text{Gumbel}(0,1)$ for independent standard Gumbel variates, with CDF $F(g) = \exp(-e^{-g})$ and density $f(g) = e^{-g}\exp(-e^{-g})$.

S2.1 The Gumbel-Max representation of categorical sampling

The forward selection rule in our algorithm,

$$J^* = \arg\max_{j \in \{1,\ldots,M\}} \left[\log a_j(\boldsymbol{X}; \boldsymbol{\theta}) + G_j\right],$$

with G_j i.i.d. standard Gumbel, samples exactly from the categorical distribution with probabilities $\pi_j = a_j/a_0$ used by the Gillespie SSA. This is the Gumbel-Max identity [8, 9]; we summarize the calculation here for completeness.

Consider first the equivalent form with normalized probabilities, $Z_j = \log \pi_j + G_j$, a standard Gumbel variate shifted by $\log \pi_j$. The shift from $\log a_j$ to $\log \pi_j = \log a_j - \log a_0$ is immaterial because argmax is invariant under additive constants common to all entries. Direct calculation shows that Z_j has the CDF

$$F_j(z) = \Pr(Z_j \le z) = \Pr(G_j \le z - \log\pi_j) = \exp(-\pi_j\, e^{-z})$$

and the corresponding density $f_j(z) = \pi_j e^{-z}\exp(-\pi_j e^{-z})$. Reaction j is selected exactly when Z_j exceeds every Z_k with $k \neq j$, so conditioning on $Z_j = z$ and using the independence of the Gumbel draws,

$$\Pr(J^* = j) = \int_{-\infty}^{\infty} f_j\,(z) \prod_{k \neq j} F_k\,(z)\,\mathrm{d}z = \int_{-\infty}^{\infty} \pi_j\; e^{-z}\exp\left(-e^{-z}\sum_{k=1}^{M} \pi_k\right)\mathrm{d}z.$$

Because $\sum_k \pi_k = 1$, the integrand collapses to the standard Gumbel density, which integrates to one, so $\Pr(J^* = j) = \pi_j$ — exactly the categorical distribution required by the Gillespie algorithm.

S2.2 Convergence of the temperature-controlled softmax to the argmax

The Gumbel-Softmax surrogate used in the backward pass replaces the hard one-hot sample $\boldsymbol{y} = \boldsymbol{e}_{J^*}$ with the temperature-controlled softmax

$$\sigma_T(\boldsymbol{z})_j = \frac{\exp(z_j/T)}{\sum_k \exp\,(z_k/T)},$$

applied to the same Gumbel-perturbed log-propensities $z_j = \log a_j + G_j$ that drive the forward selection. The justification for this replacement is that as $T \to 0^+$, the softmax collapses onto the hard argmax. Therefore, at low temperature, the surrogate is close to the object it stands in for. The collapse is exponentially fast, which is what makes the surrogate useful in practice.

Let $z_{(1)} \ge z_{(2)}$ denote the two largest components of $\boldsymbol{z}$, and let $j^* = \mathrm{argmax}_j z_j$ (the maximum is unique with probability one under the continuous Gumbel distribution). Writing $\Delta_k = z_{(1)} - z_k \ge 0$, with $\Delta_{j^*} = 0$ and $\Delta_k \ge z_{(1)} - z_{(2)}$ for $k \neq j^*$, the softmax weight on the maximum is

$$\sigma_T(\boldsymbol{z})_{j^*} = \frac{1}{1 + \sum_{k \neq j^*} \exp\,(-\Delta_k/T)}.$$

Each term in the sum satisfies $\exp(-\Delta_k/T) \le \exp(-(z_{(1)} - z_{(2)})/T)$, so the gap between $\sigma_T(\boldsymbol{z})_{j^*}$ and one satisfies

$$0 \le 1 - \sigma_T(\boldsymbol{z})_{j^*} \le (M-1)\exp\big(-(z_{(1)} - z_{(2)})/T\big),$$

which decays exponentially in $1/T$. Applied to the Gumbel-perturbed log-propensities $z_j = \log a_j(\boldsymbol{X}; \boldsymbol{\theta}) + G_j$, this yields the Gumbel-Softmax relaxation of categorical sampling [9, 10]: as $T \to 0^+$, $\sigma_T(\boldsymbol{z}) \to \boldsymbol{e}_{J^*}$ almost surely, where J^* is the Gumbel-Max sample of Section S2.1.

S2.3 Statistical exactness of the forward pass

Fix any temperature $T > 0$. The straight-through construction produces forward trajectories that are statistically identical to those of the standard Gillespie SSA, regardless of the temperature used in the backward pass.

The argument has three pieces. First, the forward update uses only the hard sample $\boldsymbol{y} = \boldsymbol{e}_{J^*}$. The soft sample $\tilde{\boldsymbol{y}} = \sigma_T(\boldsymbol{z})$ enters only through the backward pass via the stop-gradient construction so

that the temperature parameter never affects which state the simulator transitions to. Second, by the Gumbel-Max identity established in Section S2.1, the firing reaction J^* has the categorical distribution with probabilities $\pi_j = a_j/a_0$, matching the SSA. Third, the waiting time $\tau = -\log(U)/a_0$ with $U \sim \mathrm{Uniform}(0,1)$ is exponential with rate a_0 by the inverse-transform method and depends on a Uniform draw disjoint from the Gumbel draws used for reaction selection, which makes reaction selection and waiting time independent, as required by the SSA. (If $a_0 = 0$, the state is absorbing and no further reaction or waiting time is drawn; the rule above applies on the non-absorbing set.) Combining the three, every step of the algorithm draws from the correct joint distribution over (firing reaction, waiting time), and the resulting sample path $\{\boldsymbol{X}(t_k)\}_{k\geq 0}$ is a sample of the underlying CTMC for every $T > 0$.

This is the formal sense in which the framework decouples physical exactness from differentiability. The backward pass uses the temperature-controlled softmax $\sigma_T(\boldsymbol{z})$ as a surrogate for the hard categorical sample (the construction in Section 2.3 of the main text), and the resulting straight-through gradient is a biased surrogate for the true gradient of the expected loss. The softmax Jacobian $\partial\sigma_T(\boldsymbol{z})/\partial\boldsymbol{z}$ has worst-case operator norm of order $1/T$ (with the explicit bound $1/(2T)$ established by Popoviciu's inequality on the categorical covariance), so very low temperatures can produce poorly conditioned or high-variance gradient estimates, even though the forward simulation remains exact. In practice the bias is controlled by temperature annealing and the variance by ensemble averaging across K parallel trajectories; the empirical bias-variance behavior across four decades of T is documented in Figure S1. The point of the forward exactness result is that none of this affects the simulator. Parameters optimized with the straight-through gradient are validated against the exact CTMC dynamics by construction, eliminating the simulation-reality mismatch present in soft-forward approaches.

Note S3: Reversible Dimerization

The reversible dimerization system consists of three molecular species and two reactions representing the formation and dissociation of a dimer from two monomers [11].

Species. Let $X_A, X_B, X_C \in \mathbb{Z}_{\geq 0}$ denote the copy numbers of species A, B, and C respectively.

Reactions. The system comprises a forward dimerization reaction and a reverse dissociation reaction:

$$A + B \xrightarrow{k_1} C$$

$$C \xrightarrow{k_2} A + B$$

Propensity functions. Under mass-action kinetics, the propensities are

$$a_1(\boldsymbol{X}) = k_1 X_A X_B$$

$$a_2(\boldsymbol{X}) = k_2 X_C$$

where k_1 and k_2 are the forward and reverse rate constants, respectively.

Stoichiometry. The stoichiometric vectors describing the change in copy numbers upon each reaction firing are

$$\boldsymbol{\nu}_1 = \begin{pmatrix} -1 \\ -1 \\ +1 \end{pmatrix}, \qquad \boldsymbol{\nu}_2 = \begin{pmatrix} +1 \\ +1 \\ -1 \end{pmatrix}$$

Conservation laws. The system satisfies two conservation relations: $X_A(t) + X_C(t) = X_A(0) + X_C(0)$ and $X_B(t) + X_C(t) = X_B(0) + X_C(0)$ for all $t \geq 0$.

Parameters. The ground truth parameters used for inference validation were $k_1 = 0.01$ and $k_2 \in \{0.01, 0.02, 0.04, 0.08, 0.16, 0.32, 0.64, 1.28\}$, with initial conditions $X_A(0) = 100$, $X_B(0) = 90$, and $X_C(0) = 0$.

Note S4: Genetic Oscillator

The Vilar et al. oscillator [12] is a genetic regulatory network exhibiting sustained oscillations through coupled positive and negative feedback loops. The model was originally proposed as a minimal circadian rhythm generator.

Species. The system comprises nine molecular species:

Symbol	Description
D_A	Activator gene (inactive state)
D_A'	Activator gene (active state)
D_R	Repressor gene (inactive state)
D_R'	Repressor gene (active state)
M_A	Activator mRNA
M_R	Repressor mRNA
A	Activator protein
R	Repressor protein
C	Activator-repressor complex

Reactions. The network comprises sixteen reactions organized into functional modules.

Gene activation and deactivation:

$$D_A + A \xrightarrow{\gamma_A} D_A'$$

$$D_A' \xrightarrow{\theta_A} D_A + A$$

$$D_R + A \xrightarrow{\gamma_R} D_R'$$

$$D_R' \xrightarrow{\theta_R} D_R + A$$

Transcription:

$$D_A' \xrightarrow{\alpha_A'} D_A' + M_A$$

$$D_A \xrightarrow{\alpha_A} D_A + M_A$$

$$D_R' \xrightarrow{\alpha_R'} D_R' + M_R$$

$$D_R \xrightarrow{\alpha_R} D_R + M_R$$

Translation:

$$M_A \xrightarrow{\beta_A} M_A + A$$

$$M_R \xrightarrow{\beta_R} M_R + R$$

Degradation:

$$M_A \xrightarrow{\delta_{M_A}} \varnothing$$

$$M_R \xrightarrow{\delta_{M_R}} \varnothing$$

$$A \xrightarrow{\delta_A} \varnothing$$

$$R \xrightarrow{\delta_R} \varnothing$$

Complex formation and dissociation:

$$A + R \xrightarrow{\gamma_C} C$$

$$C \xrightarrow{\theta_C} R$$

Propensity functions. All reactions follow mass-action kinetics.

Reference parameters. The nominal parameter values producing sustained oscillations are: $\gamma_A = 1.0$, $\theta_A = 50.0$, $\gamma_R = 1.0$, $\theta_R = 100.0$, $\alpha_A' = 50.0$, $\alpha_A = 0.5$, $\alpha_R' = 500.0$, $\alpha_R = 0.01$, $\beta_A = 50.0$, $\beta_R = 5.0$, $\delta_{M_A} = 10.0$, $\delta_{M_R} = 0.5$, $\delta_A = 1.0$, $\delta_R = 0.2$, $\gamma_C = 2.0$, $\theta_C = 1.0$.

Inferred parameters. The five parameters selected for inference were the translation rates β_A and β_R, the protein degradation rates δ_A and δ_R, and the complex formation rate γ_C.

Note S5: Gene Regulatory Network for Classification

The classification network implements a two-layer gene regulatory architecture using a thermodynamic model of promoter occupancy. An input image [13] provides a fixed transcription factor field that regulates hidden genes, which in turn regulate output genes corresponding to digit classes.

Species and inputs. The model comprises three types of molecular entities:

Symbol	Dimension	Description
T	$\mathbb{R}_{\geq 0}^{784}$	Input transcription factors (fixed per image)

Symbol	Dimension	Description
H	$\mathbb{Z}_{\geq 0}^{256}$	Hidden gene products
O	$\mathbb{Z}_{\geq 0}^{10}$	Output gene products

The input transcription factor concentrations T_p for $p = 1, \ldots, 784$ are set equal to the normalized pixel intensities of the input image and held constant during simulation.

Thermodynamic promoter model. Each gene has a promoter with two coarse-grained states (OFF and ON). The ON-state carries a Boltzmann weight $\exp(E)$ where E is a dimensionless activation energy, while the OFF-state has weight 1 [14]. The probability of the ON state is therefore

$$P(\text{ON} \mid E) = \frac{\exp(E)}{1 + \exp(E)} = \sigma(E)$$

where $\sigma(\cdot)$ denotes the logistic sigmoid function. Activation energies are modeled as affine functions of regulator activities.

Hidden layer regulation. For each hidden gene H_i with $i = 1, \ldots, 256$, the activation energy is

$$E_i^{(H)}(T) = b_i^{(H)} + \sum_{p=1}^{784} W_{pi}^{(H)} T_p$$

where $W^{(H)} \in \mathbb{R}^{784\times256}$ and $b^{(H)} \in \mathbb{R}^{256}$ are learnable parameters. The production propensity is

$$a_i^{(H,\text{prod})}(T) = k_{\max} \cdot \sigma\big(E_i^{(H)}(T)\big)$$

with maximal production rate $k_{\max} = 2.0$. Hidden gene products degrade via first-order kinetics:

$$a_i^{(H,\text{deg})}(H_i) = \gamma_i^{(H)} H_i$$

where $\gamma_i^{(H)} > 0$ is a learnable degradation rate constrained to be positive via softplus transformation plus a positive constant that serves as a tunable lower bound for the degradation rate.

Output layer regulation. Output genes are regulated by the hidden genes products copy numbers H_i. For each output gene O_j with $j = 1, \ldots, 10$, the activation energy is

$$E_j^{(O)}(H) = b_j^{(O)} + \sum_{i=1}^{256} W_{ij}^{(O)} H_i$$

where $W^{(O)} \in \mathbb{R}^{256\times10}$ and $b^{(O)} \in \mathbb{R}^{10}$ are learnable parameters. The production and degradation propensities are

$$a_j^{(O,\text{prod})}(H) = k_{\max} \cdot \sigma\big(E_j^{(O)}(H)\big)$$

$$a_j^{(O,\text{deg})}(O_j) = \gamma_j^{(O)} O_j$$

where $\gamma_i^{(O)} > 0$ is a learnable degradation rate constrained to be positive via softplus transformation plus a positive constant that serves as a tunable lower bound for the degradation rate.

Reaction network. The complete model defines a continuous-time Markov jump process with 532 reactions:

Hidden layer (512 reactions): For $i = 1, \dots, 256$:

$$\varnothing \xrightarrow{a_i^{(H,\text{prod})}} H_i \quad \text{and} \quad H_i \xrightarrow{a_i^{(H,\text{deg})}} \varnothing$$

Output layer (20 reactions): For $j = 1, \dots, 10$:

$$\varnothing \xrightarrow{a_j^{(O,\text{prod})}} O_j \quad \text{and} \quad O_j \xrightarrow{a_j^{(O,\text{deg})}} \varnothing$$

Learnable parameters. The complete parameter set θ comprises:

Parameter	Shape	Count
$W^{(H)}$	784×256	200,704
$b^{(H)}$	256	256
$W^{(O)}$	256×10	2,560
$b^{(O)}$	10	10
$\gamma^{(H)}$	256	256
$\gamma^{(O)}$	10	10
Total		203,796

Classification readout. Given an input image with fixed transcription factor field T, the network is trained for 2,880 reaction events. The predicted digit is the index of the output gene with highest copy number at termination:

$$\hat{y} = \arg\max_{j \in \{0,\dots,9\}} O_j$$

Training objective. During training, the output copy numbers are converted to probabilities via softmax and the loss is categorical cross-entropy against one-hot encoded labels. This differentiable relaxation enables gradient-based optimization while the readout interpretation remains the biochemically natural winner-take-all on molecule counts.

Note S6: Ion Channel Gating Kinetics

The ion channel model describes conformational transitions of voltage-gated sodium channels recorded via patch-clamp electrophysiology [15]. Unlike the chemical reaction systems above, this model represents conformational changes of individual macromolecules rather than reactions, demonstrating the generality of the framework to a general continuous-time Markov chain.

Species. The system tracks the number of channels in each conformational state:

Symbol	Description
n_C	Number of channels in closed state

Symbol	Description
n_O	Number of channels in open state
n_I	Number of channels in inactivated state

The total number of channels is conserved: $n_C + n_O + n_I = 2$.

Reactions. The kinetic scheme comprises three transitions:

Index	Reaction	Propensity	Description
1	C → O	$a_1 = k_{\text{open}} \cdot n_C$	Channel opening
2	O → C	$a_2 = k_{\text{close}} \cdot n_O$	Channel closing
3	O → I	$a_3 = k_{\text{inact}} \cdot n_O$	Inactivation (irreversible)

Stoichiometry matrix. The state change vectors are arranged as rows:

$$\mathbf{S} = \begin{pmatrix} -1 & +1 & 0 \\ +1 & -1 & 0 \\ 0 & -1 & +1 \end{pmatrix}$$

where columns correspond to species (n_C, n_O, n_I) and rows to reactions (opening, closing, inactivation).

Absorbing state. The inactivated state I is absorbing: once a channel enters state I, it remains there indefinitely. This is appropriate for the experimental timescale, as recovery from inactivation requires repolarization and occurs on a timescale of tens to hundreds of milliseconds—far longer than the 8 ms recording window. When all channels are inactivated (state [0,0,2]), all propensities are zero.

Initial conditions. All simulations begin with all channels in the closed state: $(n_C, n_O, n_I) = (2,0,0)$. This matches the experimental protocol where channels recover to the closed state during the inter-sweep interval at hyperpolarized holding potential.

Training procedure. Rate constants are parameterized in log-space to ensure positivity: $k_i = \exp(\theta_i)$. The loss function minimizes the mean squared error between simulated and experimental ensemble-averaged open channel counts:

$$\mathcal{L}(\boldsymbol{\theta}) = \frac{1}{T}\sum_{t=1}^{N}\left(\langle n_O\rangle_{\text{sim}}(t_i;\boldsymbol{\theta}) - \langle n_O\rangle_{\text{exp}}(t_i)\right)^2$$

where N is the number of experimental timepoints and $\langle n_O\rangle_{\text{sim}}$ is estimated by Monte Carlo averaging over $M = 262{,}144$ parallel stochastic simulations.

Training hyperparameters:

Hyperparameter	Value
Simulations per gradient step	262,144
Gillespie steps per simulation	20
Training epochs	400

Hyperparameter	Value
Optimizer	RMSprop
Learning rate	0.05 (annealed)
Total training time	112 s

Validation. After training, learned parameters were validated using 30,000 independent exact Gillespie simulations (without any relaxation), achieving R^2 = 0.987, RMSE = 0.021 open channels, and NRMSE = 3.5%.

Data availability. Single-channel recordings are publicly available at Zenodo: https://zenodo.org/records/7817601. File used: HEK293_Cell01_40mV_100s_raw_DET_IDEl.xlsx.

Note S7: GPU throughput benchmark

We measured the throughput of three SSA implementations: standard, Gumbel-Max, and Gumbel-Softmax. All results are end-to-end wall-clock throughput measured in SSA steps/sec, where one step is one Gillespie event (one CTMC jump).

Platform. Linux 5.15.0-151-generic; Python 3.11.13; TensorFlow 2.20.0 (GPU, XLA). CPU: AMD EPYC 7343 (32 cores). GPU: NVIDIA RTX 6000 Ada Generation (49 GB), driver 570.124.06; cuDNN 9.3.0.

Workload. Ensembles of $N \in \{1, 10, 100, 10^3, 10^4, 10^5, 10^6\}$ independent trajectories. Each run uses 10 blocks × 5,000 steps/block = 50,000 SSA steps/trajectory. Throughput is computed as

$$\text{steps/sec} = \frac{N \times 10 \times 5000}{T_{\text{total}}}.$$

Key results (saturated regime).

Num trajectories	Method	Steps/sec	Relative vs standard
100,000	Standard	2,295,096,927	1.00×
100,000	Gumbel-Softmax	1,930,666,170	0.84×
100,000	Gumbel-Max	1,366,311,550	0.60×
1,000,000	Standard	2,997,688,090	1.00×
1,000,000	Gumbel-Softmax	1,890,659,846	0.63×
1,000,000	Gumbel-Max	1,396,688,262	0.47×

Across tested ensemble sizes, Gumbel-Softmax achieves ~0.63–0.93× the throughput of the standard simulator (batch-size dependent), while Gumbel-Max shows larger overhead at the largest ensemble in this configuration (~0.47× at 10^6 trajectories).

Supporting References

1. Williams, R.J. (1992). Simple statistical gradient-following algorithms for connectionist reinforcement learning. Machine Learning *8*, 229-256.
2. Gupta, A., and Khammash, M. (2014). An efficient and unbiased method for sensitivity analysis of stochastic reaction networks. Journal of The Royal Society Interface *11*, 20140979.
3. Rathinam, M., Sheppard, P.W., and Khammash, M. (2010). Efficient computation of parameter sensitivities of discrete stochastic chemical reaction networks. The Journal of Chemical Physics *132*, 034103.
4. Bengio, Y., Léonard, N., and Courville, A. (2013). Estimating or propagating gradients through stochastic neurons for conditional computation. arXiv:1308.3432.
5. Tucker, G., Mnih, A., Maddison, C.J., Lawson, J., and Sohl-Dickstein, J. (2017). Rebar: Low-variance, unbiased gradient estimates for discrete latent variable models. Advances in Neural Information Processing Systems *30*, 2624–2633.
6. Mohamed, S., Rosca, M., Figurnov, M., and Mnih, A. (2020). Monte Carlo gradient estimation in machine learning. J. Mach. Learn. Res. *21*, Article 132.
7. Rijal, K., and Mehta, P. (2025). A differentiable Gillespie algorithm for simulating chemical kinetics, parameter estimation, and designing synthetic biological circuits. eLife *14*, RP103877.
8. Gumbel, E.J. (1954). Statistical theory of extreme values and some practical applications: a series of lectures, Volume 33, (US Government Printing Office).
9. Maddison, C.J., Mnih, A., and Teh, Y.W. (2017). The Concrete distribution: A continuous relaxation of discrete random variables. In International Conference on Learning Representations.
10. Jang, E., Gu, S., and Poole, B. (2017). Categorical reparameterization with Gumbel-Softmax. In International Conference on Learning Representations.
11. Kampen, N.G.v. (2007). Stochastic processes in physics and chemistry, 3rd Edition, (Amsterdam ; Boston: Elsevier).
12. Vilar, J.M.G., Kueh, H.Y., Barkai, N., and Leibler, S. (2002). Mechanisms of noise-resistance in genetic oscillators. Proc Natl Acad Sci U S A *99*, 5988-5992.
13. LeCun, Y., Bottou, L., Bengio, Y., and Haffner, P. (1998). Gradient-based learning applied to document recognition. Proceedings of the IEEE *86*, 2278-2324.
14. Vilar, J.M.G., and Saiz, L. (2013). Systems biophysics of gene expression. Biophys J *104*, 2574-2585.
15. Selimi, Z., Rougier, J.-S., Abriel, H., and Kucera, J.P. (2023). A detailed analysis of single-channel Nav1.5 recordings does not reveal any cooperative gating. The Journal of Physiology *601*, 3847-3868.

Supporting Figures

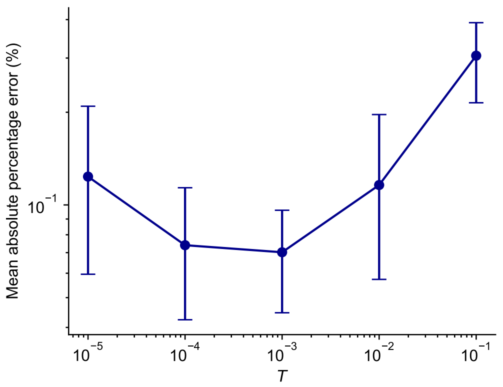

Figure S1. Robustness of dimerization parameter recovery to the Gumbel-Softmax annealing target. The inference protocol from Section 3.1 of the main text was applied with geometric annealing from $T = 1.0$ to $T \in \{10^{-1}, 10^{-2}, 10^{-3}, 10^{-4}, 10^{-5}\}$ to each of the 8 ground-truth conditions of Figure 2 ($k_1 = 0.01$ fixed, $k_2 \in \{0.01, 0.02, 0.04, 0.08, 0.16, 0.32, 0.64, 1.28\}$). Every other element of the protocol was held identical to the main run. Markers show the mean MAPE across the 8 ground-truth conditions; error bars show 95% bootstrap confidence intervals (20,000 resamples). Across four decades of the final temperature T (10^{-5}– 10^{-2}) the average MAPE remains within approximately 50% of its minimum (0.070%–0.124).

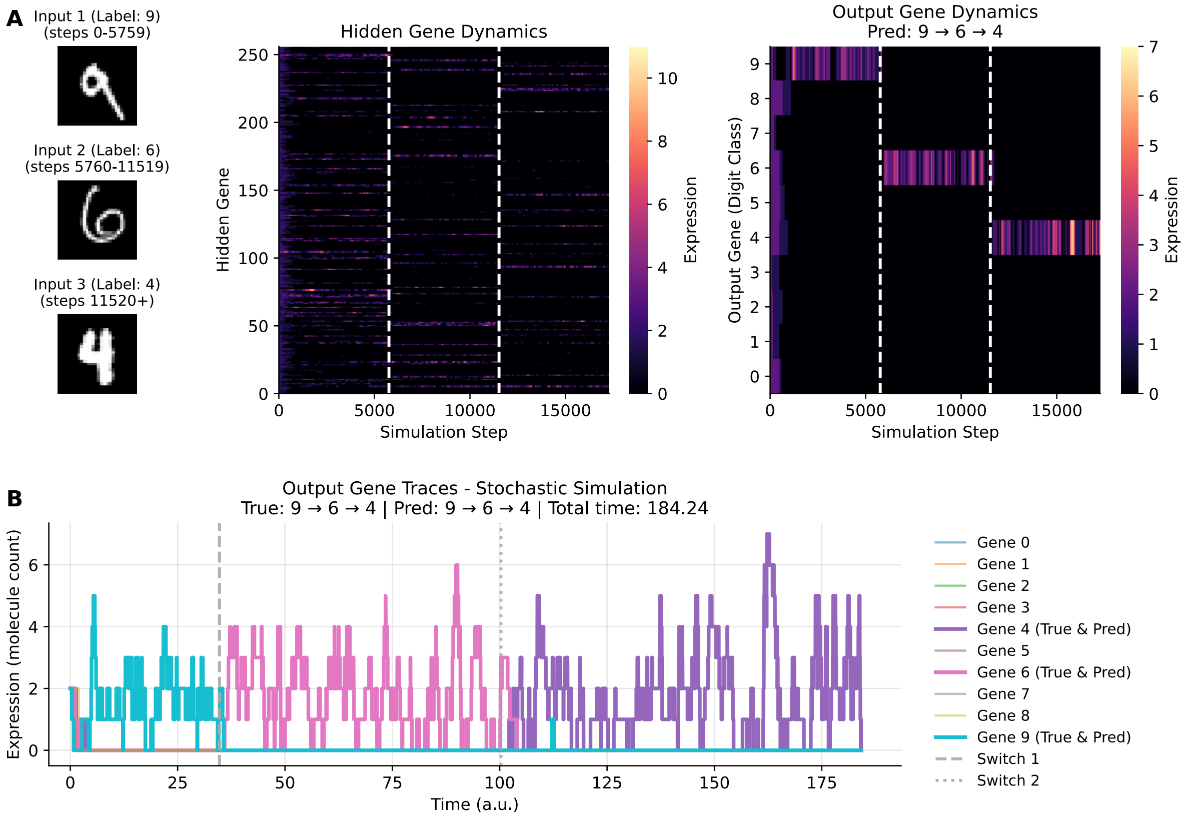

Figure S2. Stochastic dynamics of the gene regulatory network during sequential digit classification with a degradation rate lower bound of 1.0. Same format, input sequence, and network architecture as Figure 5 (digits 9, 6, and 4), but using the model trained with a higher degradation rate (lower bound = 1.0; see Figure 4F). (**A**) (**Left**) Input images presented sequentially: digit "9" (simulation steps 0–5,759), digit "6" (steps 5,760–11,519), and digit "4" (steps 11,520+). (**Middle**) Hidden gene dynamics. Heatmap showing expression levels (molecule counts) for all 256 hidden genes over approximately 18,000 simulation steps. Dashed vertical lines indicate input image transitions. Despite the higher degradation rate, which increases molecular turnover and stochastic noise, distinct subsets of hidden genes activate in response to each digit. (**Right**) Output gene dynamics. Heatmap showing expression levels for the 10 output genes corresponding to digit classes 0–9. The network correctly classifies all three digits (Pred: 9 → 6 → 4). (**B**) Output gene time traces from the stochastic simulation. Continuous-time expression traces for all 10 output genes, with genes corresponding to correct predictions highlighted: Gene 9 (cyan), Gene 6 (pink), and Gene 4 (purple). Dashed vertical lines mark input switching times. The correct output gene achieves dominant expression during each input phase, demonstrating that the network maintains accurate classification even under elevated degradation, albeit with increased stochastic fluctuations in gene expression levels compared to the lower degradation rate regime (Figure 5). True sequence: 9 → 6 → 4; Predicted sequence: 9 → 6 → 4.

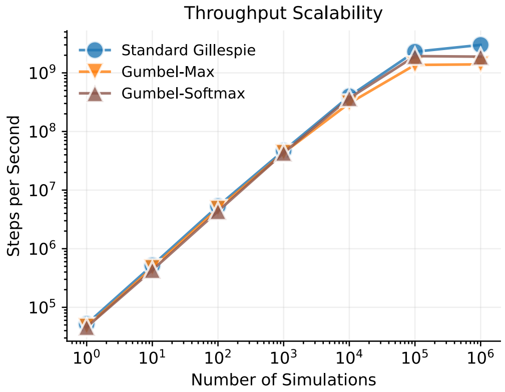

Figure S3. Computational throughput as a function of ensemble size. Simulation throughput (steps per second) was measured for the Vilar oscillator system using three reaction selection methods: Standard Gillespie with categorical sampling (blue circles), Gumbel-Max with argmax selection (orange inverted triangles), and Gumbel-Softmax with temperature-controlled soft sampling (brown triangles). All methods show near-linear scaling with ensemble size from 1 to 10^6 parallel simulations. At large ensemble sizes, throughput reaches approximately 10^9 steps per second, with all three methods achieving comparable performance. The near-identical throughput across methods demonstrates that the differentiable framework incurs minimal computational overhead compared to non-differentiable implementations. Benchmarks performed on an NVIDIA RTX 6000 Ada Generation GPU with TensorFlow 2.20.

Supporting Algorithm

Algorithm S1. Differentiable exact stochastic simulation via forward/backward decoupling.

Inputs:

- Parameter vector $\boldsymbol{\theta} \in \mathbb{R}^p$
- Initial state $\boldsymbol{X}_0 \in \mathbb{Z}^N$
- Stoichiometry vectors $\{\boldsymbol{\nu}_j\}_{j=1}^{M}$ and propensity functions $\{a_j(\boldsymbol{X};\boldsymbol{\theta})\}_{j=1}^{M}$
- Loss functional $\ell(\boldsymbol{X}_{0:n}, t_{0:n})$ over a length-n trajectory
- Ensemble size K, trajectory length n, temperature schedule $\{T_e\}_{e=1}^{E}$, learning-rate schedule $\{\eta_e\}_{e=1}^{E}$

Output: Optimized parameter vector $\boldsymbol{\theta}^*$.

for epoch $e = 1, \dots, E$ **do**
    Set $T \leftarrow T_e, \eta \leftarrow \eta_e$
    Initialize K parallel trajectories: $\boldsymbol{X}^{(i)} \leftarrow \boldsymbol{X}_0$, $t^{(i)} \leftarrow 0$ for $i = 1, \dots, K$
    Open gradient tape on $\boldsymbol{\theta}$
    for step $k = 1, \dots, n$ **do**   ▷ *forward pass — exact CTMC*
        Compute $a_j^{(i)} \leftarrow a_j(\boldsymbol{X}^{(i)};\boldsymbol{\theta})$ for all i, j
        Draw $U^{(i)} \sim$ Uniform(0,1); set $\tau^{(i)} \leftarrow -\log U^{(i)} / a_0^{(i)}$ ▷ *waiting time*
        Draw $G_j^{(i)} \overset{\text{i.i.d.}}{\sim}$ Gumbel(0,1)
        Form $z_j^{(i)} \leftarrow \log a_j^{(i)} + G_j^{(i)}$
        $\boldsymbol{y}^{(i)} \leftarrow$ one_hot$(\text{argmax}_j z_j^{(i)})$ ▷ *Gumbel-Max selection*
        $\widetilde{\boldsymbol{y}}^{(i)} \leftarrow$ softmax$(\boldsymbol{z}^{(i)}/T)$ ▷ *temperature-controlled surrogate*
        $\boldsymbol{y}_{\text{ST}}^{(i)} \leftarrow$ stopgrad$(\boldsymbol{y}^{(i)} - \widetilde{\boldsymbol{y}}^{(i)}) + \widetilde{\boldsymbol{y}}^{(i)}$
        $\boldsymbol{X}^{(i)} \leftarrow \boldsymbol{X}^{(i)} + \sum_{j=1}^{M} y_{\text{ST},j}^{(i)} \boldsymbol{\nu}_j;\ \ t^{(i)} \leftarrow t^{(i)} + \tau^{(i)}$
    end for
    $\mathcal{L} \leftarrow \frac{1}{K}\sum_{i=1}^{K} \ell\,(\boldsymbol{X}_{0:n}^{(i)}, t_{0:n}^{(i)})$
    $\nabla_{\boldsymbol{\theta}}\mathcal{L}$ via automatic differentiation   ▷ *backward pass through* $\widetilde{\boldsymbol{y}}^{(i)}$
    $\boldsymbol{\theta} \leftarrow$ Optimizer$(\boldsymbol{\theta}, \nabla_{\boldsymbol{\theta}}\mathcal{L}, \eta)$
end for
return $\boldsymbol{\theta}^* \leftarrow \boldsymbol{\theta}$
